# Strong Father–Child Relationships and Other Positive Childhood Experiences, Adverse Childhood Experiences, and Sexual Risk Factors for HIV among Young Adults Aged 19–24 Years, Namibia, 2019: A Cross-Sectional Study

**DOI:** 10.3390/ijerph20146376

**Published:** 2023-07-16

**Authors:** Nickolas T. Agathis, Francis B. Annor, Likang Xu, Elizabeth Swedo, Laura Chiang, Rachel Coomer, Jennifer Hegle, Pragna Patel, Norbert Forster, Gabrielle O’Malley, Alison L. Ensminger, Rahimisa Kamuingona, Helena Andjamba, Brigitte Nshimyimana, Molisa Manyando, Greta M. Massetti

**Affiliations:** 1 Epidemic Intelligence Service, Centers for Disease Control and Prevention, Atlanta, GA 30333, USA; 2 Division of Violence Prevention, National Center for Injury Prevention and Control, Centers for Disease Control and Prevention, Atlanta, GA 30341, USA; 3 Division of Global HIV and TB, Global Health Center, Centers for Disease Control and Prevention, Private Bag, Windhoek 12029, Namibia; 4 Division of Global HIV and TB, Global Health Center, Centers for Disease Control and Prevention, Atlanta, GA 30333, USA; 5 International Training and Education Center for Health, Department of Global Health, University of Washington, Seattle, WA 98195, USA; 6 Ministry of Gender Equality, Poverty Eradication, and Social Welfare, Private Bag, Windhoek 13359, Namibia; 7 US Agency for International Development, Washington, DC 20004, USA

**Keywords:** HIV, sexual health, adolescent and young adult health, adverse childhood experiences, positive childhood experiences, fathers

## Abstract

Using cross-sectional data from the 2019 Namibia Violence Against Children and Youth Survey and sex-stratified multivariable models, we assessed the associations between four different positive childhood experiences (PCEs) and having ≥3 adverse childhood experiences (ACEs), including ≥3 ACE–PCE interaction terms, and seven sexual risk factors for HIV acquisition among young adults aged 19–24 years. One PCE, having a strong father–child relationship, was inversely associated with two risk factors among women (lifetime transactional sex (OR, 0.4; 95% CI, 0.2–0.7) and recent age-disparate sexual relationships (OR, 0.3; 95% CI, 0.2–0.5)), and significantly interacted with having ≥3 ACEs for three risk factors among women (not knowing a partner’s HIV status, infrequently using condoms, and ever having an STI) and one among men (having multiple sexual partners in the past year). The other PCEs were significantly associated with ≤1 HIV risk factor and had no significant interaction terms. Strong father–child relationships may reduce HIV acquisition risk and mitigate the effect of childhood adversity on HIV risk among young adults in Namibia.

## 1. Introduction

Reducing the HIV burden and incidence among adolescents and young adults, particularly girls and young women, given their disproportionate risk in high-HIV-incidence countries, is essential to curbing the global HIV epidemic [1]. In 2022, 350,000 adolescents and young adults aged 15–24 were newly infected globally [2]. In Namibia, where HIV/AIDS is the number one cause of death and disability (measured using DALYs, or disability-adjusted life years) among the general population (as of 2019) [3], adolescents and young adults aged 15–24 accounted for approximately 19% of the population, 12% of all people living with HIV, and 37% of new HIV infections annually in 2022 [4]. Understanding what factors influence the risk of HIV acquisition among adolescents and young adults, especially girls and young women, and implementing interventions that target these factors can help improve their well-being and reduce the overall HIV burden and incidence in Namibia.

Heterosexual intercourse is a key mode of HIV transmission and has helped propagate the HIV epidemics in Namibia and other sub-Saharan African countries [5]. Sexual risk factors associated with HIV acquisition include behaviors such as engaging in transactional sex [6,7], infrequently using condoms [8], having multiple sexual partners [9,10], not knowing a sexual partner’s HIV status [11], and having age-disparate sexual relationships with substantially older partners [12,13]. Other sexual HIV risk factors, including sexual-health-related outcomes, are also independently associated with HIV acquisition, such as having a non-HIV STI [14,15] and experiencing sexual violence in adulthood [16].

Earlier experiences in childhood and adolescence may influence the sexual HIV risk later in life. Adverse childhood experiences (ACEs) are potentially traumatic events that occur in childhood and can increase the risks of adverse health behaviors and poor health outcomes, including HIV acquisition and associated sexual risk factors, in adolescents and adults [17]. Studies have demonstrated an association between ACEs and sexual HIV risk factors in adulthood, including sexual risk behaviors [18,19,20,21], having STIs [22,23], and experiencing sexual violence in adulthood [24]; however, most of these studies did not disaggregate by sex. Data on sex-specific associations between ACEs and sexual HIV risk factors are limited but important because young men and women may respond to ACEs differently [25] and carry different HIV risk profiles [26] that can significantly influence the interventions that may help reduce their HIV acquisition risk. Furthermore, published data on the prevalence and impact of ACEs and their association with the HIV acquisition risk are generally lacking in many sub-Saharan African countries, including Namibia.

In contrast to ACEs, positive childhood experiences (PCEs), which include factors like safe, stable, and nurturing relationships and environments [27], can reduce the risk of adverse health behaviors and poor health outcomes in adolescents and adults [28,29]. PCEs not only may be protective against adverse health outcomes but also mitigate the harmful effects of ACEs on long-term health among those who have experienced childhood adversity [30,31,32,33]. Most of the evidence on PCEs is related to their impact on mental health; however, studies have subsequently demonstrated beneficial effects on sexual health [34,35,36]. PCEs, strong parental relationships, and secondary school attendance and completion are associated with reduced levels of sexual risk behaviors among young adults in sub-Saharan Africa [34,35,36]. More evidence is needed to understand the sex-specific influence of PCEs on HIV sexual risk and how PCEs may mitigate the effect of ACEs on HIV sexual risk among young men and women.

This cross-sectional analysis aims to understand how exposure to ACEs and PCEs may influence the occurrence of different sexual HIV risk factors among young adults in Namibia. This analysis has four objectives: (1) to compare the prevalence of six ACEs, four PCEs, and seven sexual HIV risk factors between young men and women; (2) to assess the sex-stratified associations between having ≥3 ACEs and the occurrence of each sexual HIV risk factor; (3) to assess the sex-stratified multivariable associations between exposure to each PCE and the occurrence of each sexual HIV risk factor; and (4) to assess if and how having each PCE moderates or potentially mitigates the sex-stratified association between having ≥3 ACEs and each sexual HIV risk factor. Understanding these associations and relationships can help inform policy and programmatic efforts to reduce the risk of HIV acquisition among adolescents and young adults in high-burden settings and aid HIV epidemic control.

## 2. Materials and Methods

### 2.1. Background and Objectives of the Namibia VACS

This study was a secondary analysis of the 2019 Namibia Violence Against Children and Youth Survey, or Namibia VACS. The Namibia VACS is a cross-sectional, nationally representative, geographically clustered multistage household survey of adolescents and young adults aged 13 to 24 years [37]. The standard VACS design and methodology have been described elsewhere [38]. Eligible participants for the Namibia VACS included females and males who lived in selected households in Namibia, were between 13 and 24 years old, and spoke Afrikaans, English, Khoekhoegowab, Oshiwambo, Otjiherero, RuKwangali, or siLozi. Children unable to participate due to severe intellectual or physical disability (hearing or speech impairments) were excluded. Trained interviewers administered standardized questionnaires face-to-face to the head of each household and male and female participants [39]. The participant questionnaires inquired about the following topics: demographics, parental and familial relationships, and education status; lifetime and recent exposures to physical, emotional, and sexual violence; disclosure of violence experiences; knowledge and utilization of services; witnessing violence in the home or community; and risk-taking behaviors and health outcomes. In addition to the interviews, voluntary HIV testing was offered to participants 14 years and older in accordance with the Namibia Ministry of Health and Social Services (MoHSS) guidelines [40].

### 2.2. Sampling of the Namibia VACS

The Namibia VACS was led by the Government of Namibia, specifically the Ministry of Gender Equality, Poverty Eradication and Social Welfare (MGEPESW); the Ministry of Health and Social Services (MoHSS); and the Namibia Statistics Agency (NSA), with financial support from the President’s Emergency Plan for AIDS Relief (PEPFAR) and implementation and technical support from the US Centers for Disease Control and Prevention (CDC) and the International Training and Education Center for Health (I-TECH) at the University of Washington (UW). The survey design followed a three-stage clustered sampling approach that included random selection of geographic sampling units or enumeration areas, households in each sampling unit, and an eligible individual in each household. A split-sample approach, in which different sampling units were selected for male and female participants to prevent retaliatory violence between opposite-sex perpetrators and the survivors of their violence, was also followed [41,42]. This approach also yielded separate estimates for males and females. The Namibia VACS was implemented nationwide; however, adolescent girls and young women were oversampled in certain regions with high HIV prevalence, specifically Khomas, Oshikoto, and Zambezi, to provide regional estimates that would inform HIV and violence prevention programming in these regions.

### 2.3. Ethical Procedures

Namibia’s MoHSS Research Ethics Committee and the CDC’s Institutional Review Board reviewed and approved this study protocol. For participants under 18, permission was obtained from a parent or guardian, as well as assent from the participant. For participants 18 years or older and minors 16 years and older who had children, were married under civil law, or were heads of households, informed consent was obtained directly from the participants.

In addition to the split-sample approach, strategies to protect participant privacy, confidentiality, and safety included voluntary, private, and one-on-one interviews. Additionally, in accordance with the WHO guidelines [42], the VACS was presented as a general youth health and wellness survey to everyone, including during the informed consent process with the heads of household and caregivers of the participants; only the participants and interviewers were aware that the interview focused on each participant’s previous violent experiences.

All participants were provided with lists of health and social services in their local area, and specific participants who were identified to be highly vulnerable were offered direct referrals to social workers through the MGEPESW. For those identified to be in acute danger, immediate action plans were enacted. The United Nations International Children’s Emergency Fund (UNICEF) supported the development, training, and implementation of the response plans through case management processes. For those who were diagnosed with HIV during the survey, the I-TECH and the MoHSS coordinated immediate linkage with HIV treatment and care services.

### 2.4. Study Sample

This secondary analysis of data from the 2019 Namibia VACS included all young men and women who participated in the Namibia VACS and who were aged 19–24 years at the time the survey was conducted. The age of 19 years was chosen as the cut-off to help ensure that childhood exposures (ACEs and PCEs) occurred before most of the outcomes and sexual HIV risk factors occurred, particularly those that were assessed to have occurred in the previous 12 months.

### 2.5. Outcomes

Seven dichotomous sexual HIV risk factors, or risk factors related to sexual health and associated with HIV acquisition, were included in this analysis:

(1) Unknown HIV status of a recent sexual partner in the past 12 months—the participant had at least one sexual partner in the past 12 months whose HIV status was unknown to the participant, i.e., the participant both denied ever having been tested with the partner and denied that the partner ever disclosed his or her HIV status;

(2) Lifetime transactional sex—the participant reported ever having sex in exchange for material support or things that he or she “needs such as money, gifts or other things that are important;”

(3) Infrequent condom use in the past 12 months—the participant reported only sometimes or never using condoms with at least one sexual partner in the past 12 months, excluding those participants who both reported being married or living with someone as if being married and reported only having one sexual partner in the past 12 months;

(4) Multiple sexual partners in the past 12 months—the participant reported having more than one sexual partner in the past 12 months;

(5) Age-disparate sexual relationship in the past 12 months—the participant reported having a sexual relationship in the past 12 months with a partner who was older than the participant by 10 or more years;

(6) STI in lifetime—the participant reported ever being diagnosed with a sexually transmitted infection or ever having a genital sore or ulcer;

(7) Sexual violence during adulthood—participant reported experiencing sexual violence at or after the age of 18 years.

### 2.6. Demographics

Three sociodemographic variables were included in this analysis: (1) age—a dichotomous variable including ages 19–21 and 22–24; (2) region of residence—a 4-level variable including Khomas, Zambezi, Oshikoto, and other regions; and (3) financial insecurity—a dichotomous variable distinguishing those who reported not having enough money for either food, “the most important things such as clothing, school fees, or medical care”, or both from those who reported having enough money for all these items.

### 2.7. Exposures—ACEs

Six dichotomous ACE measures that would have occurred before 18 years of age were included in this analysis:

(1) Experiencing physical violence, defined as if an intimate partner; a peer; a parent, an adult caregiver, a relative, or another adult in the community did any of the following:

(a) Slapping, pushing, shoving, shaking, or intentionally throwing something at the participant;
(b) Punching, kicking, whipping, or beating the participant with an object;
(c) Strangling, smothering, intentionally burning, or trying to drown the participant;
(d) Threatening or hurting the participant with a knife, a panga, a gun, or another weapon.

(2) Experiencing emotional violence, defined as any of the following:

(a) If a parent, an adult caregiver, or another adult relative (i) told the participant he or she was not loved or did not deserve to be loved; (ii) said that they wished the participant had never been born or were dead; or (iii) ridiculed or put the participant down;
(b) If an intimate partner (i) insulted, humiliated, or made fun of the participant in front of others; (ii) kept the participant from having money; (iii) tried to keep the participant from seeing or talking to family or friends; (iv) kept track of the participant by demanding to know where he or she has been and what he or she has been doing; or (v) made threats to physically harm the participant;
(c) If a peer (i) made participant get scared or feel really bad because peer was calling participant names, saying mean things, or saying they didn’t want participant around; (ii) told lies or spread rumors about participant; (ii) told lies or spread rumors about the participant; or (iii) kept the participant out of things on purpose, excluded the participant from their group of friends, or completely ignored the participant.

(3) Experiencing sexual violence, defined as any of the following:

(a) Being touched in a sexual way without the participant’s permission, including “fondling, pinching, groping, or touching” on or around his or her sexual body parts;
(b) Being physically forced to have sex against his or her will, when sex did occur;
(c) Being physically forced to have sex, when sex did not occur;
(d) Being pressured to have sex through harassment and threats, when sex did occur.

(4) Witnessing physical violence in the community, defined as witnessing someone being attacked outside the home;

(5) Witnessing physical violence in the home, defined as witnessing a father or step-father hit, punch, or kick a mother or step-mother or vice versa, or witnessing a parent punch, kick, or beat siblings;

(6) Becoming an orphan, with at least one parent deceased prior to the participant becoming 18 years old.

A cumulative ACE score or indicator was also calculated for each participant and included two groups—those who experienced 0, 1, or 2 ACEs and those who experienced ≥3 ACEs. These two groups were chosen to compare the associations between high levels of adversity and lower levels of adversity in childhood. Two groups were chosen instead of three groups (e.g., 0 ACEs, 1–2 ACEs, and ≥3 ACEs) to facilitate interpretation of the interaction between PCEs and ACEs and understand the potential role PCEs have in moderating the association between high levels of adversity and sexual HIV risk factors.

### 2.8. Exposures—PCEs

Four dichotomous PCEs measures were included in this analysis:

(1) Attending or completing secondary school, in which the participant reported having attended or completed secondary school by the time of the survey;

(2) Having a strong mother–child relationship (under 18);

(3) Having a strong father–child relationship (under 18);

(A strong father/mother–child relationship is defined as a participant reporting that, while he or she was under 18, the participant both had an easy time talking to his or her biological father/mother and was close to his or her biological father/mother.)

(4) Strong caregiver monitoring and supervision, in which the participant reported that the person he or she had the closest relationship with (either mother, father, or other caregiver) knew “a lot” about at least one of the following while the participant was under 18: (a) who the participant’s friends were; (b) how the participant spent money; (c) where the participant went after school; (d) where the participant went at night; or (e) what the participant did in his or her free time.

### 2.9. Data Analysis

First, the prevalence of the demographic variables, sexual HIV risk factors, ACEs, and PCEs was estimated and compared among males and females using chi-square tests (*p*-values < 0.05). Second, bivariate analyses were conducted to calculate crude odds ratios (ORs) that assessed the associations between the sexual HIV risk factors and the individual ACEs and PCEs and the cumulative ACE scores, i.e., having ≥3 ACEs compared to 0–2 ACEs, among men and women (*p*-value < 0.05). Third, multivariable logistic regression analyses were used to assess the association between having ≥3 ACEs (compared to 0–2 ACEs) and each sexual HIV risk factor, adjusted for demographics (age, region of residence, and financial insecurity).

Fourth, multivariable logistic regression analyses assessed the association between each PCE and each sexual HIV risk factor. In this step, for each sexual HIV risk factor (dependent variable), four models were constructed. Each model included an ACE variable, the three demographic variables, one of the four PCEs, and an interaction term between the cumulative ACE indicator and the PCE under consideration. All multivariable analyses were stratified by sex; the determination to stratify this way was made a priori to assess the sex-based differences in the associations. This hierarchical analytic approach was taken rather than combining all the ACEs and PCEs into one model due to limitations in the sample size. For all multivariable analyses, a Bonferroni correction was applied and alpha = 0.01 was used instead of 0.05 (five models were used for each of the ACE–sexual risk factor associations). Fifth, for all interaction terms that had *p*-values < 0.01 in the previous step, stratified multivariable analyses were conducted, if the sample size permitted, to assess the associations between having ≥3 ACEs (compared to 0–2 ACEs) and the HIV sexual risk factors, stratified by those with and those without the PCE under consideration.

This analysis was performed using SAS (version 9.4; SAS Institute), accounting for the complex survey design (including survey weight, cluster, and strata).

## 3. Results

### 3.1. Descriptive Analysis

Of the 4211 girls and young women who completed the Namibia VACS (89% overall response rate), 2046 (49%) were 19–24 years old and eligible for this analysis, and of the 980 boys and young men who completed the VACS (84% overall response rate), 467 (48%) were 19–24 years old and eligible for this analysis. The sociodemographic characteristics were similar among the men and the women. Over half of the eligible young adults were aged 19–21 years. Over two-thirds (68%) of the young adults experienced financial insecurity, i.e., not having enough money for food and/or other essential items like schooling or clothes (Table 1).

Not having known the HIV status of at least one sexual partner and having used condoms infrequently in the past 12 months were common sexual HIV risk factors (24% and 29%, respectively) among all young adults in Namibia aged 19–24 years. Significantly more young women than men used condoms infrequently in the past 12 months (38% vs. 21%, *p* < 0.001) and had age-disparate sexual relationships with partners who were older by 10 years or more in the past 12 months (3.7% vs. 0.4%, *p* < 0.001). Conversely, more young men than women did not know the HIV status of at least one sexual partner in the past 12 months (29% vs. 18%, *p* < 0.001) and had more than one sexual partner in the past 12 months (25% vs. 7%, *p* < 0.001, Table 1).

Approximately 79% of the Namibian young adults in this study had at least one ACE and 26% had three or more ACEs, with no significant differences between the men and the women. Significantly more women, however, experienced sexual violence than men (11% vs. 6%, *p* < 0.05), whereas significantly more men experienced physical violence (39% vs. 31%, *p* < 0.05) and witnessed physical violence in the community (60% vs. 49%, *p* < 0.05, Table 2). The prevalence of experiencing emotional violence (9%), witnessing violence in the home (37%), and being an orphan (29%) was not significantly different between the men and the women. Attending or completing secondary school was the most prevalent PCE among the men and the women (86%). The women were more likely than the men to have attended or completed secondary school (91% vs. 82%, *p* < 0.01), while the men were more likely than the women to have strong father–child relationships (55% vs. 43%, *p* < 0.01; Table 2).

### 3.2. Bivariate Association between ACEs, PCEs, and Sexual HIV Risk Factors

Experiencing three or more ACEs, compared to experiencing 0–2 ACEs, was significantly associated with five sexual HIV risk factors among the men and four risk factors among the women (Table S1). Completing or attending secondary school was significantly associated with two sexual HIV risk factors among the women and one risk factor among the men. Having a strong father–child relationship was significantly associated with four sexual HIV risk factors among the women and two risk factors among the men. Having a strong mother–child relationship was significantly associated with one sexual HIV risk factor among women and three risk factors among men. Strong caregiver monitoring and supervision were significantly associated with three sexual HIV risk factors among the women and one risk factor among the men (Table S2). Bivariate associations could not be assessed for age-disparate sexual relationships among the men, given the low prevalence of this risk factor among men.

### 3.3. Multivariable Associations between Cumulative ACE Score, Individual PCEs, and Sexual HIV Risk Factors

After adjustment for demographic variables, having ≥3ACEs, compared to having 0–2 ACEs, was significantly associated with three of the seven sexual HIV risk factors among the women―lifetime transactional sex (OR, 4.2; 95% CI, 2.0–8.7; Table 3), having age-disparate sexual relationships in the past 12 months (OR, 2.0; 95% CI, 1.2–3.3), and experiencing sexual violence in adulthood (OR, 2.9; 95% CI, 1.7–5.1). Among the men, having ≥3 ACEs was only significantly associated with not knowing partners’ HIV statuses (OR, 2.1; 95% CI, 1.4–3.1; Table 4). Multivariable associations could not be determined for three sexual HIV risk factors among the men—lifetime transactional sex, having age-disparate sexual relationships in the past 12 months, and experiencing sexual violence in adulthood—given the limited prevalence of these reported risk factors among men and the failure of the models to converge.

After adjustment for demographics and having ≥3 ACEs, several PCE and sexual HIV risk factor associations were statistically significant for both the young men and women. Among the women, having a strong father–child relationship was significantly associated with decreased odds of lifetime transactional sex (OR, 0.4; 95% CI, 0.2–0.7) and decreased odds of having recent age-disparate sexual relationships (OR, 0.3; 95% CI, 0.2–0.5); experiencing strong caregiver monitoring and supervision was associated with increased odds of ever having STIs (OR, 3.0; 95% CI, 1.6–5.7); and attending or completing secondary school was significantly associated with decreased odds of having age-disparate sexual relationships in the past 12 months (OR, 0.2; 95% CI, 0.1–0.5; Table 3). Among the men, having a strong mother–child relationship was significantly associated with decreased odds of ever having an STI (OR, 0.4; 95% CI, 0.2–0.8; Table 4).

The interaction between having strong father–child relationships and having ≥3 ACEs was statistically significant for four of the sexual HIV risk factors—three among the women (Table 3) and one among the men (Table 4)—and required stratified analyses (Table 5). Among the women who did not have strong relationships with their fathers in childhood, experiencing ≥3 ACEs was associated with increased odds of not knowing a partner’s HIV status in the past 12 months (OR, 1.7; 95% CI, 1.7–2.4), increased odds of infrequently using condoms in the past 12 months (OR, 1.4; 95% CI, 1.0–2.1), and increased odds of ever having STIs (OR, 2.3; 95% CI, 1.2–4.6). Conversely, among the women who had strong relationships with their fathers in childhood, these associations were nullified or inverted—experiencing ≥3 ACEs was associated with decreased odds of not knowing a partner’s HIV status in the past 12 months (OR, 0.6; 95% CI, 0.3–1.0), decreased odds of infrequently using condoms in the past 12 months (OR, 0.6; 95% CI, 0.4–0.9), and decreased odds of ever experiencing STIs (OR, 0.3; 95% CI, 0.1–1.0). Among the men who did not have strong relationships with their fathers in childhood, experiencing ≥3 ACEs was associated with increased odds of having multiple sexual partners (OR, 3.5; 95% CI, 1.9–6.4), while among men who did have strong relationships with their fathers, experiencing ≥3 ACEs was associated with decreased odds of having multiple sexual partners (OR, 0.9; 95% CI, 0.4–1.9).

## 4. Discussion

Our analysis of the 2019 Namibia VACS data suggests that strong parent–child relationships, particularly father–daughter relationships, may be protective and mitigate the negative impact that high levels of childhood adversity can have on HIV acquisition risk among young adults aged 19–24 in Namibia. Having a strong father–child relationship was found to be protective against ever having transactional sex and having recent age-disparate sexual relationships among women, and having a strong mother–child relationship was protective against being diagnosed with STIs among men. Furthermore, having a strong father–child relationship moderated the association between high levels of ACEs and four of the seven sexual HIV risk factors in this analysis: three among women and one among men.

These findings are novel, as few studies have assessed how strong parental relationships can mitigate the effects of ACEs on HIV sexual risk. Evidence has clearly demonstrated, however, that social protection and support, including strong parental support, prevent adolescent participation in behaviors associated with HIV risk and decrease HIV acquisition risk [36,43]. Some studies have linked the specific benefits of paternal support to reducing the risk of having many sexual partners over the course of a lifetime or multiple partners in the past 12 months [36,44]. A study from South Africa demonstrated that having a father in the home moderated and mediated the effects of school-based intervention aimed at reducing the risk of sexual intercourse in the previous three months and unprotected intercourse among adolescents. Further work is needed to explore these associations and understand the mechanisms linking ACEs, strong relationships with parents and other primary caregivers, and HIV acquisition risk [45].

Understanding which childhood experiences (e.g., strong paternal relationships) benefit young adults and protect them from the negative effects of ACEs is critical, given the strong effect of ACEs on HIV acquisition risk. Indeed, our analysis detected significant associations between having ≥3 ACEs and increased odds of several sexual HIV risk factors among both men and women. The Namibia VACS has clearly shown that childhood adversity is a common experience among young adults in Namibia. Nearly three out of four young women and over four out of five men in Namibia have had ≥1 ACE, and one of four young adults has had ≥3 ACEs. Studies from other African countries have also reported consistently high prevalence of experiencing ≥1 ACE, ranging from 75 to 99% [19,46,47,48,49,50]. In our analysis, the prevalence of cumulative ACEs did not differ significantly by sex, but the prevalence of individual ACEs did. Previous studies from both outside and within sub-Saharan Africa have also corroborated our findings that girls and young women are more likely to have experienced sexual violence in childhood and boys and young men may be more likely to have been exposed to community violence; on the other hand, while we demonstrated that young men and boys are more likely to have experienced physical violence, the findings from these previous studies were mixed [19,46,48,49,51,52,53].

Next steps should be considered to strengthen the evidence and better understand the negative effects of ACEs and positive effects of PCEs on the occurrence of sexual HIV risk factors in young adults in sub-Saharan Africa. Surveys examining violence against children could be expanded to incorporate more comprehensive information on ACEs and PCEs (as long as ethical and methodological requirements are met to ensure that the risk of harm to the participant from asking violence-related questions is minimized) [42]. Regarding ACEs, surveys of violence among children, like the VACS, could expand measurement of household challenges in childhood, especially parent and family member health and circumstances during childhood. Regarding PCEs, the recent literature has attempted to create frameworks to holistically capture experiences in childhood that may lead to resilience. These new comprehensive approaches to understanding positive experiences, like the HOPE framework and the Benevolent Childhood Experiences scale, could influence future VACS questionnaire items [28,54]. Future VACSs could also include more questions about caregivers other than biological parents to assess whether these child–caregiver relationships are potential PCEs.

Other analytic approaches, like multi-country analyses using the VACS, could also help us to further understand the association between ACEs and HIV risk and inform global HIV prevention efforts. Such analyses could leverage both VACS questionnaire items related to HIV and voluntary HIV testing data to assess other, rarer outcomes that more directly assess HIV risk, like new or recent HIV diagnoses. Future studies may consider replicating this study using VACS data from other countries and other data sources to assess if the results observed in this study hold: particularly the potentially mitigating effect of parental relationships on the association between ACEs and HIV risk factors. Such efforts should aim to include relatively larger samples of male participants, such as oversampling males in high-HIV-prevalence regions.

Our findings could have significant programmatic and policy implications. They support the application of violence prevention strategies, using technical packages like INSPIRE, to improve the well-being of children, adolescents, and young adults in Namibia and other sub-Saharan African countries and reduce their HIV risk. INSPIRE is a set of seven evidence-based strategies recommended by the WHO and its partners to help end violence against children [55]. Our findings have specifically highlighted the importance of improving parent and caregiver support, one of the INSPIRE strategies, which aims to reduce harsh parenting practices and create close parent–child relationships by emphasizing non-violent discipline and close and effective communication [55]. Several programs have been implemented in Namibia and other countries, intending to fulfill this strategy and help create strong parent–child relationships. For example, the Families Matter program is a community-based group intervention focused on improving parental knowledge, skills, and self-efficacy in communicating with adolescents about sexuality, safe sex practices, sexual violence, and ways to reduce their sexual and HIV risks. Evaluations of the program in Tanzania and Kenya have demonstrated increased and improved communication about sexual issues between adolescents and parents who completed the program [55,56]. Parenting for Lifelong Health, or “Sinovuyo Teen”, is a group-based parenting program that includes both parents and their adolescents and emphasizes positive parenting using social learning principles. Studies of the program in South Africa have demonstrated that it is cost-effective and associated with several outcomes, including improved positive parenting and discipline and reduced rates of childhood violence, poor mental health among caregivers and adolescents, and caregiver substance abuse [57,58,59].

Our findings also support the cross-disciplinary combination-prevention approach of DREAMS (Determined, Resilient, Empowered, AIDS-free, Mentored, and Safe). DREAMS is a PEPFAR-supported partnership that implements evidence-based interventions aimed to help prevent HIV among adolescent girls and young women (AGYWs). It was first implemented in Khomas, Zambezi, and Oshikoto in 2017, leading to VACS oversampling in those regions, and since has expanded to other high-incidence areas in the country. DREAMS focuses on reducing the risk among both AGYWs and their sexual partners, strengthening families through parenting and caregiver programs, and mobilizing communities for change [60]. In Namibia, DREAMS has partnered with the in-country NGO Lifeline/Childline Namibia to implement positive parenting training programs like Families Matter! to parents of DREAMS participants, strengthen parent–adolescent relationships, and reduce HIV risk for AGYWs [61].

### Limitations

Our analysis benefitted from the Namibia VACS’s large and nationally representative sample; its comprehensive and expert-based questionnaire; and its rigorous survey methodology that has promoted privacy, confidentiality, and accuracy in its responses. Nevertheless, the limitations of the survey may hinder interpretation of our findings. First, recall and social desirability bias may have influenced our findings and altered the observed associations from the actual ones. Second, the cross-sectional data in this analysis cannot permit causal inferences between the exposure—ACEs and PCEs—and the outcomes—sexual HIV risk factors. Third, the present findings apply to youth living in households; these results cannot be generalized to children and young adults who are institutionalized, are homeless, or were unable to complete the VACS due to disability. Fourth, the Namibia VACS did not collect data on all potential ACEs, and therefore, our analysis may have underestimated the number of children exposed to other ACEs and the number of ACEs to which children in Namibia are exposed. Fifth, besides the question assessing caregiver support and monitoring, the VACS currently does not collect substantive data on the roles of key secondary caregivers in a child’s life, including biological relatives like grandparents or unrelated caregivers like neighbors, family friends, or adopted parents. Sixth, the smaller sample size of male participants may have limited the power to detect significant associations in men; multivariable analyses could not be carried out for three of the seven risk factors among men. Seventh, while those in same-sex relationships, particularly gay, bisexual, and other men who have sex with men, have an increased risk of HIV acquisition, the VACS did not inquire about the history or characteristics of same-sex relationships.

Three additional limitations exclusively related to this analysis may also have hindered the interpretation of our findings. First, our analysis may have been prone to type 1 statistical errors given the numerous associations assessed, though our analysis used a Bonferroni correction to mitigate this limitation. Furthermore, this report’s main findings are supported by consistent associations among multiple sexual HIV risk factors; these multiple significant associations are not very likely to all be due to statistical error. Second, variables other than demographics not included in this analysis due to sample size considerations may have confounded, mediated, or moderated associations and influenced our findings. For example, the mental health of young adults may be an important mediator that has influenced the relationship between childhood experiences and HIV risk in young adulthood. Poor mental health has been shown to be associated with high levels of both ACEs and HIV risk factors among adolescents and young adults in Sub-Saharan Africa [46,62,63,64]. Meinck et al. showed, in their longitudinal study that followed adolescent girls aged 12-17 years in South Africa, that mental health distress completely mediated the relationship between ACEs at baseline and increased HIV risk behaviors (including infrequent condom use in the past year, lifetime sex while intoxicated, and more than two sexual partners in the past year) at a 1-year follow-up [35]. Parental experience of ACEs may also be another important confounder in these relationships. Due to increased stress, parents who have experienced ACEs may not be able to control their stress systems or their parenting styles [65]. As a result, the intergenerational effects of ACEs might be transmitted through common pathways such as parental mental health and unfavorable parenting practices [66].

Lastly, to optimally assess the association of ACEs and PCEs with HIV risk, using new HIV infections or incidences as the outcome would have been optimal, and the VACS did collect data on HIV infection statuses. However, these HIV infection data were not used for two primary reasons. First, the HIV testing was offered voluntarily and was not standardized (therefore, the VACS data may not have accurately estimated the actual HIV prevalence, and there may have been confounding by indication; i.e., whether a participant was tested or not would confound the analysis). Second, the HIV infection data that were collected from the VACS could only assess the prevalence and not the incidence of HIV infection; we could not determine with these data whether each participant recently acquired HIV or acquired it through vertical transmission before or at birth or early in life. Therefore, the HIV infection data collected could not validly assess risk.

## 5. Conclusions

This study suggests that youth in Namibia who have had strong parent–child relationships, particularly father–daughter relationships, may have reduced likelihoods of experiencing various HIV acquisition risk factors. In addition, we found that PCEs like strong parent–child relationships may mitigate the effect that childhood adversity has on the likelihood that these youth would experience these HIV acquisition risk factors. While further research is needed, this study supports the important role strong parent–child relationships may have by building resilience; promoting positive health, particularly sexual health; and protecting many young adults from the negative health effects of the adversity that they have experienced in childhood. Understanding the role of these positive parental relationships and implementing socioecological comprehensive strategies that include evidence-based positive parenting programs are critical to controlling the HIV epidemic and improving the well-being of children, adolescents, and young adults in Namibia.

## Figures and Tables

**Table 1:** Demographics and sexual HIV risk factors among young adults, 19–24 years old, in Namibia.

Indicator	Female		Male		Total	
	N	Weighted Prevalence % (95% CI)	N	Weighted Prevalence % (95% CI)	N	Weighted Prevalence % (95% CI)
**Demographic**						
Age	2046		467		2513	
19–21		55.0 (51.5–58.6)		57.8 (51.8–63.8)		56.5 (52.9–60.1)
22–24		45.0 (41.4–48.5)		42.2 (36.2–48.2)		43.5 (39.9–47.1)
Region of Residence	2046		467		2513	
Khomas		21.6 (16.5–26.6)		16.2 (6.7–25.8)		18.7 (14.3–23.1)
Oshikoto		10.5 (7.9–13.1)		9.2 (0.5–17.8)		9.8 (5.5–14.1)
Zambezi		5.6 (4.3–7.0)		4.8 (0.0–10.2)		5.2 (2.5–7.9)
Other		62.3 (54.3–70.3)		69.8 (57.5–82.1)		66.3 (60.8–71.7)
Financial Insecurity	2012	63.7 (57.9–69.5)	464	71.8 (65.4–78.3)	2476	68.0 (63.8–72.3)
**Outcomes**						
Did Not Know HIV Status of at Least One Sexual Partner in the past 12 Months *	1972	18.1 (14.9–21.3)	445	29.3 (23.7–34.9)	2417	24.0 (20.6–27.5)
Ever Had Transactional Sex	1999	3.4 (2.1–4.7)	462	2.6 (1.2–4.1)	2461	3.0 (2.0–4.0)
Multiple Sexual Partners in the past 12 Months *	1975	7.1 (5.1–9.2)	445	24.6 (20.2–29.1)	2420	16.4 (13.2–19.6)
Infrequent or no Condom Use in the Past 12 Months *	1961	38.2 (34.1–42.3)	442	21.2 (17.0–25.5)	2403	29.2 (25.6–32.8)
Sexual Partner in the Past 12 Months Who Was Older by ≥10 years *	1889	3.7 (2.2–5.3)	435	0.4 (0.0–0.8)	2324	1.9 (1.1–2.8)
History of STI in Lifetime	2034	7.2 (5.2–9.2)	467	9.0 (5.6–12.4)	2501	8.1 (5.9–10.4)
Experienced Sexual Violence in Adulthood (18 Years Old to Present)	2027	9.6 (7.2–12.0)	464	7.3 (3.5–11.0)	2491	8.3 (6.0–10.7)

* Chi-square *p*-value < 0.001. N = unweighted sample size in sex-stratum or total sample.

**Table 2:** Adverse and positive childhood experiences (ACEs and PCEs, respectively) among young adults, 19–24 years old, in Namibia.

Indicator	Female		Male		Total	
	N	Weighted Prevalence % (95% CI)	N	Weighted Prevalence % (95% CI)	N	Weighted Prevalence % (95% CI)
**ACEs (Before Age of 18 Years)**						
Experienced Physical Violence *	2015	30.6 (26.4–34.8)	453	39.4 (32.7–46)	2468	35.3 (31.6–39)
Experienced Sexual Violence *	2032	10.9 (8.3–13.5)	467	6.1 (3.3–9.0)	2499	8.3 (6.4–10.3)
Experienced Emotional Violence	1977	10.6 (7.8–13.4)	458	7.0 (4.6–9.3)	2435	8.7 (7.0–10.3)
Witnessed Physical Violence in the Community *	1997	49.1 (44.7–53.5)	451	60.2 (52.5–67.9)	2448	55 (50.2–59.8)
Witnessed Physical Violence at Home	1991	35.9 (31.5–40.3)	449	38.8 (31.8–45.8)	2440	37.4 (33.5–41.4)
Orphan Status	1915	32.5 (27.7–37.4)	446	26.4 (21.3–31.5)	2361	29.3 (25.7–32.8)
≥1 ACEs	2037	77.2 (73.3–81.1)	467	81.0 (75.3–86.7)	2504	79.2 (75.7–82.7)
≥3 ACEs	2037	25.5 (21.4–29.5)	467	26 (19.9–32)	2504	25.7 (22.4–29.1)
**PCEs (Before Age of 18 Years)**						
Education Status: Completed or Attended Secondary School **	2032	90.6 (87.7–93.5)	467	81.8 (76–87.7)	2499	85.9 (82.4–89.4)
Strong Mother–Child Relationship (Before 18 Years)	1950	68.0 (64.0–72.1)	461	74.7 (69.0–80.5)	2411	71.7 (67.9–75.5)
Strong Father–Child Relationship (Before 18 Years) **	1864	42.5 (37.0–48)	447	55.3 (49.6–61.0)	2311	49.6 (45.7–53.4)
Caregiver Monitoring and Supervision (Before 18 Years)	2002	64.0 (59.5–68.4)	459	67.3 (60.4–74.1)	2461	65.7 (61.5–70.0)

* *p*-value < 0.05. ** *p*-value < 0.01. N = unweighted sample size in sex-stratum or total sample size.

**Table 3:** Multivariable associations between sexual HIV risk factors, ≥3 ACEs, and individual PCEs among young women.

	Adjusted OR (CI 95%)						
	Did Not Know Partner’s HIV Status in the Past 12 Months	Lifetime Transactional Sex	Multiple Sexual Partners in the Past 12 Months	Infrequent Condom Use in the Past 12 Months	Lifetime STI	SV at 18 Years or Older	Sexual Partner in the Past 12 Months Who Was Older by ≥10 Years
Exposure							
ACEs 3 or More (ref: <3 ACEs) ^1^	1.1 (0.8–1.5)	4.2 (2.0–8.7) ***	2.2 (1.2–4.0) *	1.0 (0.6–1.7)	1.5 (0.8–2.8)	2.9 (1.7–5.1) ***	2.0 (1.2–3.3) **
Attended or Completed Secondary School ^2^	0.6 (0.4–0.9) *	0.6 (0.2–1.8)	0.7 (0.2–2.1)	0.8 (0.4–1.6)	0.6 (0.2–1.5)	0.6 (0.3–1.2)	0.2 (0.1 0.5) ***
Strong Relationship with Mother ^2^	0.9 (0.6–1.4)	0.8 (0.4–1.6)	0.5 (0.3–0.9) *	0.9 (0.6–1.3)	0.9 (0.6–1.5)	0.8 (0.5–1.3)	0.5 (0.2–1.4)
Strong Relationship with Father ^2^	n/a ^3^	0.4 (0.2–0.7) **	1.5 (1.0–2.2)	n/a ^3^	n/a ^3^	0.9 (0.6–1.4)	0.3 (0.2–0.5) ***
Caregiver Monitoring and Supervision ^2^	1.2 (0.9–1.5)	1.5 (0.5–4.0)	1.4 (0.9–2.0)	0.8 (0.6–1.0) *	3.0 (1.6–5.7) **	2.3 (1.2–4.6) *	0.8 (0.3–2.1)

* *p*-value < 0.05. ** *p*-value < 0.01. *** *p*-value < 0.001. ^1^ Adjusted for age, region of residence, and financial insecurity. ^2^ Adjusted for age, region of residence, financial insecurity, and experience of three or more ACEs. ^3^ Interaction term for PCE variable and variable of three or more ACEs is statistically significant (*p*-value < 0.01).

**Table 4:** Multivariable associations between sexual HIV risk factors, ≥3 ACEs, and individual PCEs among young men.

	Adjusted OR (95% CI)						
	Did Not Know Partner’s HIV Status in Past 12 Months	Lifetime Transactional Sex	Multiple Sexual Partners in the Past 12 Months	Infrequent Condom Use in the Past 12 Months	Lifetime STI	SV at 18 Years or Older	Sexual Partner in the Past 12 Months Who Was Older by ≥10 Years
Exposure							
ACEs 3 or More (ref: <3 ACEs) ^1^	2.1 (1.4–3.1) **	n/a ^4^	1.7 (1.0–2.8)	2.2 (1.1–4.0) *	1.7 (1.2–2.6) *	n/a ^4^	n/a ^4^
Attended or Completed Secondary School ^2^	0.7 (0.5–1.0)	n/a ^4^	0.7 (0.3–1.6)	0.2 (0.1–0.6) *	0.8 (0.3–1.8)	n/a ^4^	n/a ^4^
Strong Relationship with Mother ^2^	0.8 (0.4–1.5)	n/a ^4^	0.6 (0.4–1.1)	0.7 (0.4–1.3)	0.4 (0.2–0.8) **	n/a ^4^	n/a ^4^
Strong Relationship with Father ^2^	1.0 (0.7–1.3)	n/a ^4^	n/a ^3^	1.0 (0.6–1.6)	0.7 (0.4–1.2)	n/a ^4^	n/a ^4^
Caregiver Monitoring and Supervision ^2^	0.6 (0.3–1.0)	n/a ^4^	1.1 (0.7–2.0)	1.1 (0.6–2.2)	1.1 (0.5–2.2)	n/a ^4^	n/a ^4^

* *p*-value < 0.05. ** *p*-value < 0.01. ^1^ Adjusted for age, region of residence, and financial insecurity. ^2^ Adjusted for age, region of residence, financial insecurity, and experiencing three or more ACEs. ^3^ Interaction term for PCE variable and variable of three or more ACEs is statistically significant (*p*-value < 0.01). ^4^ Sample size was too small to model.

**Table 5:** Multivariable associations between sexual HIV risk factors and ≥3 ACEs, stratified by father–child relationship status.

			Experienced ≥3 ACEs	
			OR and 95% CIs by Stratum ^2^	
			Among Those Who Had Strong Relationships with Their Fathers	Among Those Who Did Not Have Strong Relationships with Their Fathers
HIV Sexual Risk Factor	Sex	*p*-Value ^1^		
Unknown HIV Status of at Least One Sexual Partner ^3^	Female	0.002	0.5 (0.3–1.0)	1.5 (1.0–2.3) *
Infrequent Condom Use ^3^	Female	0.005	0.6 (0.4–0.9) **	1.4 (1.0–2.1)
STI in Lifetime	Female	0.005	0.3 (0.1–1.0)	2.3 (1.2–4.6) *
Multiple Sexual Partners ^3^	Male	0.004	0.9 (0.4–1.9)	3.5 (1.9–6.4) ***

* *p*-value < 0.05. ** *p*-value < 0.01. *** *p*-value < 0.001. ^1^ *p*-value for interaction term for having a strong paternal relationship) and having three or more ACEs. ^2^ Adjusted for age, region of residence, and financial insecurity. ^3^ In the previous 12 months.

## Data Availability

Data are available by request from the Together for Girls Partnership (https://www.togetherforgirls.org/request-access-vacs/ accessed on 14 July 2023).

## Supplementary Materials

The following supporting information can be downloaded at: https://www.mdpi.com/article/10.3390/ijerph20146376/s1. Table S1: Unadjusted associations between sexual HIV risk factors and ACEs among young adults in Namibia.; Table S2: Unadjusted associations between sexual HIV risk factors and PCEs among young adults in Namibia
