# Supplementary material for: Strong Father–Child Relationships and Other Positive Childhood Experiences, Adverse Childhood Experiences, and Sexual Risk Factors for HIV among Young Adults Aged 19–24 Years, Namibia, 2019: A Cross-Sectional Study
Source: Int J Environ Res Public Health. 2023 Jul 16;20(14):6376. doi: 10.3390/ijerph20146376 (PMC10378761; doi:10.3390/ijerph20146376)
Supplement: Supplementary file 1 [file ijerph-20-06376-s001.zip › ijerph-2349941-supplementary.pdf]

Supplementary Materials:

Scheme 1. Table S1: Unadjusted associations between sexual HIV risk factors and ACEs among young adults in Namibia.

		Odds Ratio (CI 95%)						
**Exposure**	**Sex**	**Don't know partner's HIV status in past 12 months**	**Lifetime transactional sex**	**Multiple sexual partners in past 12 months**	**Infrequent condom use in past 12 months**	**Lifetime STI**	**SV at 18 years or older**	**Sexual partner in the past 12 months who is older by ≥10 years**
**Experienced physical violence**	**F**	1.1 (0.9,1.5)	3.3 (1.7,6.1)***	1.9 (1.1,3.2)**	0.9 (0.6,1.4)	1.7 (1.0,2.9)	2.0 (1.2,3.5)**	2.1 (0.9, 4.9)
	**M**	2.5 (1.6,3.9)***	4.1 (1.5,11.1)**	1.9 (1.2,3)*	1.7 (1.0,2.8)*	1 (0.6,1.8)	1.5 (0.7,3.2)	n/a
**Experienced sexual violence**	**F**	1.2 (0.7,2.1),	7.0 (3.0,16.0)***	0.7 (0.3,1.5)	0.7 (0.5,1.2)	2.7 (1.5,4.8)***	8.9 (5.3,15.1)***	1.1 (0.4, 3.1)
	**M**	0.9 (0.3,2.1)	8.2 (1.6,43.2)**	2.1 (1,4.6)*	1.3 (0.4,3.6)	0.5 (0.1,2.2)	22.8 (8.7,59.4)***	n/a
**Experienced emotional violence**	**F**	0.8 (0.5,1.4)	3.1 (1.5,6.5)**	1.9 (0.9,3.8)	1.2 (0.7,2.0)	1.9 (0.9,4.0)	1.9 (0.9,4.1)	1.3 (0.2, 6.6)
	**M**	2.4 (1.3,4.7)**	4.8 (1.0,22.8)*	2.2 (1.0,4.8)*	1.0 (0.4,2.6)	1.2 (0.4,3.4)	1.0 (0.3,3.6)	n/a
**Witnessed physical violence in community**	**F**	1.0 (0.7,1.4)	5.4 (3.3,8.9)***	1.5 (0.9,2.6)	1.0 (0.8,1.3)	2.3 (1.4,4.0)**	2.5 (1.4,4.6)**	1.6 (0.9, 2.8)
	**M**	1.4 (0.8,2.6)	9.6 (5.1,17.8)***	1.5 (1,2.3)*	1.5 (0.9,2.6)	2.4 (1.1,5.5)*	6.2 (1.5,24.7)**	n/a
**Witnessed physical violence in home**	**F**	1.5 (1.1,2.0)**	2.9 (1.4,6.2)**	1.8 (1.1,3.1)*	1.1 (0.8,1.5)	2.3 (1.3,4.3)**	2 (1.1,3.7)*	2.1 (1.3, 3.4)**
	**M**	1.5 (1.0,2.2)*	3.6 (1.4,9.5)**	1.6 (1.0,2.7)	1.2 (0.7,1.8)	1.6 (0.8,3.0),	1.5 (0.7,3.1)	n/a
**Orphan status** (ref: Both parents alive at 18)	**F**	0.5 (0.3,0.8)**	0.6 (0.2,1.8)	0.8 (0.5,1.3)	1.3 (1.0,1.8)*	0.6 (0.3,1.2)	1.1 (0.6,1.8)	0.9 (0.5, 1.6)
	**M**	1.4 (0.9,2.1)	0.9 (0.4,2.1)	0.9 (0.5,1.5)	0.8 (0.5,1.3)	1.4 (0.8,2.7)	2.4 (1.2,4.7)**	n/a
**ACEs 3 or more (**ref: 0-2 ACEs**)**	**F**	1.3 (0.95-1.7)	4.4 (2.1-9.2)***	2.2 (1.2-4.2)*	1.1 (0.8-1.5)	1.8 (1.02-3.1)*	3.1 (1.7-5.5)***	1.9 (0.9, 3.9)
	**M**	2.1 (1.5-3.1)***	5.7 (2.2-14.6)***	2.1 (1.3-3.4)**	1.3 (0.8-2.1)	2.3 (1.4-3.8)**	5.1 (2.5-10.7)***	n/a

*p-value<0.05.

**p-value<0.01.

***p-value<0.001.

Scheme 2. Table S2: Unadjusted associations between sexual HIV risk factors and PCEs among young adults in Namibia.

		OR (95% CI)						
**Exposure**	**Sex**	**Don't know partner's**	**Lifetime transactional sex**	**Multiple sexual partners**	**Infrequent condom**	**Lifetime STI**	**SV at 18 years or older**	**Sexual partner in the**

		HIV status in past 12 months		in past 12 months	use in past 12 months			past 12 months who is older by ≥10 years
**Completed or attended secondary school**	**F**	0.6 (0.4,0.9)*	0.5 (0.2,1.7)	0.7 (0.3,2.0)	0.8 (0.4,1.4)	0.7 (0.3,1.7)	0.8 (0.4,1.6)	0.2 (0.1, 0.5)***
	**M**	1.0 (0.6,1.5)	0.8 (0.3,2.1)	0.9 (0.4,1.8)	0.4 (0.2,0.8)***	1.1 (0.5,2.6)	2.4 (0.6,10.2)	n/a
**Strong dad relationship**	**F**	0.9 (0.7,1.2)	0.3 (0.2,0.5)***	1.2 (0.8,1.8)	1.0 (0.7,1.3)	0.6 (0.3,0.9)*	0.8 (0.5,1.2)	0.3 (0.1, 0.5)***
	**M**	0.8 (0.5,1.3)	1.8 (0.6,5.4)	1.0 (0.5,1.7)	1.0 (0.6,1.5)	0.7 (0.4,1.2)	0.3 (0.2,0.4)***	n/a
**Strong mom relationship**	**F**	0.9 (0.6,1.4)	0.7 (0.4,1.3)	0.5 (0.3,0.8)**	0.9 (0.6,1.3)	0.8 (0.5,1.3)	0.7 (0.5,1.1)	0.5 (0.2, 1.3)
	**M**	0.7 (0.4,1.2)	0.7 (0.3,2.2)	0.6 (0.4,1.0)*	0.7 (0.4,1.3)	0.3 (0.2,0.6)***	0.3 (0.1,0.6)***	n/a
**Caregiver monitoring and supervision**	**F**	1.1 (0.8,1.4)	1.6 (0.6,4.1)	1.4 (0.9,2.1)	0.7 (0.6,0.9)*	2.4 (1.3,4.3)*	2.1 (1.1,4.0)*	0.7 (0.3, 2.0)
	**M**	0.8 (0.4,1.5)	1.2 (0.4,3.5)	1.3 (0.7,2.4)	1.2 (0.7,2.1)	1.1 (0.7,1.7)	3.2 (1.1,9.6)*	n/a

*p-value<0.05.
**p-value<0.01.
***p-value<0.001.
